# Effect of Carbon Fiber Surface Microstructure on Composite Interfacial Property Based on Image Quantitative Characterization Technique

**DOI:** 10.3390/ma14216367

**Published:** 2021-10-25

**Authors:** Shu Xiong, Yan Zhao, Jiupeng Song

**Affiliations:** School of Materials Science and Engineering, Beihang University, Beijing 100191, China; xiongshu@buaa.edu.cn (S.X.); buaajiupeng@126.com (J.S.)

**Keywords:** carbon fiber, surface microstructure, quantitative characterization, interfacial property, bonding mechanism

## Abstract

The surface roughness (Ra) and composite interfacial property of carbon fiber (CF) are considered to be mainly affected by the microstructure of the CF surface. However, quantitative characterization of the CF surface microstructure is always a difficulty. How the CF surface microstructure affects the interfacial property of CF composites is not entirely clear. A quantitative characterization technique based on images was established to calculate the cross-section perimeter and area of five types of CFs, as well as the number (N), width (W) and depth (D) of grooves on these CF surfaces. The CF composite interfacial shear strength (IFSS) was tested by the micro-droplet debonding test and modified by the realistic perimeter. The relationship between the groove structure parameter and the Ra, specific surface area and composite interfacial property was discussed in this article. The results indicated that the CF cross-section perimeter calculated by this technique showed strong consistency with the CF specific surface area and composite interfacial property. At last, the composite interface bonding mechanism based on defect capture was put forward. This mechanism can be a guiding principle for CF surface modification and help researchers better understand and establish interface bonding theories.

## 1. Introduction

Carbon fiber-reinforced polymers (CFRPs), as the important representative of advanced composites, have been extensively used in high-end manufacturing industries, such as the aeronautic and aerospace industries [1,2,3,4]. The performance of CFRPs is mainly decided by reinforcement, matrix and interface [5]. The physical and chemical properties of the CF surface are the two most important factors affecting the properties of the composite interface [6,7,8,9]. In most cases, these physical and chemical properties affect interface bonding simultaneously [10,11,12,13]. The parameters of constituent materials and interfacial properties are essential conditions for the simulation and calculation of composite properties. For example, the perimeter of carbon fiber was input into the micro-droplet debonding finite element model to calculate the interfacial strength of CF composites and this value was then compared with the tested interfacial strength [14,15]. Finite element models have also been used to design and optimize some macro composite structures [16,17].

CF is mainly manufactured by two methods: the dry-jet wet spinning process and the wet spinning process [18,19,20,21]. The surface of CF manufactured by the dry-jet wet spinning process is smooth, but the surface of CF manufactured by wet spinning is rough and full of grooves [18,22,23]. These grooves are distributed along the axial direction of CF [24,25], and their distribution on the CF surface is random. Roughness (Ra) is a parameter to describe the average fluctuation of CF surface structure. Usually, Ra is characterized by atomic force microscopy (AFM) [26,27,28,29]. However, AFM cannot scan the CF surface in 360°. Some deviations may occur in the test of Ra when the CF filament has a very irregular cross-section. More importantly, Ra cannot give out a more accurate message of all grooves. A new method for characterizing the surface microstructure of carbon fiber is urgently needed.

In recent years, a quantitative characterization method of the physical microstructure of the CF surface based on an image technique was gradually put forward by some researchers [22,30,31]. By this method, the groove parameters, including the number, width, depth and distribution of grooves, can be obtained as well as the perimeter and area of the CF cross-section. However, in their papers, they did not further explore the relationship between these parameters and the specific surface area and composite interfacial property. Interfacial shear strength (IFSS) is usually tested to evaluate the composite interfacial property by the micro-droplet debonding test [32,33,34,35]. The calculation of IFSS needs the contact area of interface. In their process of calculation, the contact area was simplified as the side surface area of an ideal cylinder, which ignored the unevenness of the CF surface. Therefore, the realistic contact area of the interface in the calculation of IFSS was considered in our study.

In this article, the surface physical microstructures of five types of CFs were characterized based on the quantitative characterization technique. Another calculation method of Ra was put forward and proved to be feasible. The IFSS of five types of CF composites was tested by the micro-droplet debonding test. Meanwhile, the IFSS was then modified by introducing the realistic perimeter of the CF cross-section. The relationships between groove parameters and the Ra, specific surface area and composite interfacial property were also explored. At last, a composite interface bonding mechanism based on defect capture was put forward and discussed.

## 2. Materials and Methods

### 2.1. Materials

Five types of polyacrylonitrile-based carbon fiber tows (CCF800, 12K, average filament diameter 5.2 μm, tensile strength about 5.4 GPa, without sizing agent) with different surface physical microstructures were supplied by Weihai Tuozhan fiber co., LTD, Weihai, China. These carbon fibers were manufactured at different solidification bath temperatures and named as CF-1, CF-2, CF-3, CF-4 and CF-5. Industrial grade epoxy resin 618 (EP618) was supplied by Zhejiang Hangtongzhou co., LTD, Jinhua, China. The hardener triethylene-tetramine was supplied by Lanyi chemical co., LTD, Beijing, China.

### 2.2. Characterization

The morphology of the CF cross-section and surface was observed by a scanning electron microscope (SEM, JSM6010, JEOL, Tokyo, Japan). The CF cross-section samples were obtained by freezing CF tows in liquid nitrogen for about 15 s and then breaking them. By this method, the most original cross-section morphologies of CF were kept. The SEM test conditions were 10.0 kV accelerating voltage, about 10 mm working distance and 5000 times and 10,000 times magnification. At least 10 CF filaments were found and photos were taken for each type of CF. In the test of CF cross-section morphology, the cross-sections must be perpendicular to the SEM lens.

CF surface Ra was characterized by AFM (Veeco D3000, Bruker Corporation, Billerica, MA, USA). At least 5 CF filaments were stuck on the glass plate one by one and then scanned in the tapping mode, with the test conditions: scanning area of 3 μm × 3 μm, frequency of 1.0 Hz. Then, CF surface Ra was calculated by NanoScope analysis software (Version 1.0.0.0.0). CF surface chemical properties were tested by X-ray photoelectron spectroscopy (XPS, AXIS UL TRA, Kratos, Manchester, UK). Brunauer–Emmet–Teller (BET) specific surface area of CF was measured on the 3H-2000PS2 analyzer (Beishide, Ltd., Beijing, China) by the method of nitrogen sorption. Before measurement, CF samples were all dried at 80 °C for 1 h.

The interfacial shear strength (IFSS) was measured by the micro-droplet debonding test machine (MODELHM410, Shinjuku, Japan). The preparation of specimens and test principle referred to our previous research work [36]. The fiber was pulled up at a rate of 0.05 mm/min until the resin micro-sphere was peeled. The length of the micro-sphere and maximum debonding load F_max_ were recorded to calculate the IFSS using Equation (1):

(1) $$\mathrm{IFSS}=\frac{F_{\max}}{\pi dL}$$

where F_max_ is the maximum debonding load, *d* is the diameter of the CF filament and *L* is the length of the CF embedded in the resin micro-sphere.

### 2.3. Quantitative Characterization of CF Surface Physical Structure

#### 2.3.1. Image Preprocessing

**Figure 1:**
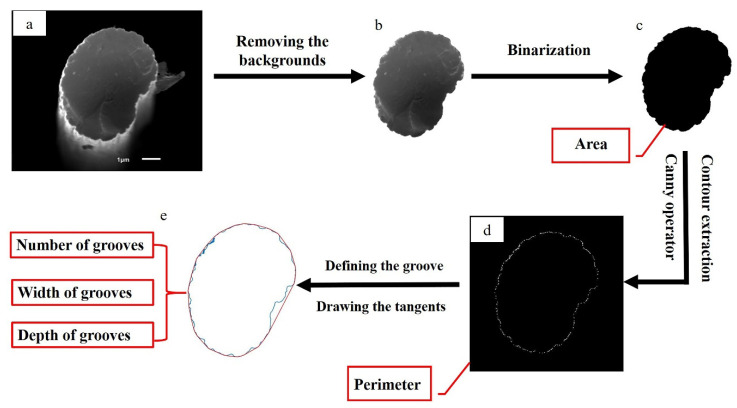
Schematic diagram of quantitative characterization process of CF surface physical structure: (**a**) Original CF cross-section SEM image, (**b**) CF cross-section SEM image without background, (**c**) CF cross-section binary image, (**d**) CF cross-section contour image, (**e**) CF cross-section edge tangent image.

The software Adobe Photoshop (version 2020) (PS) was used to preprocess the cross-section SEM image (as shown in Figure 1a) of CF. The CF cross-section boundary was automatically obtained by the above software to form a closed area. Then, the other area outside the boundary was selected and deleted totally. The original and processed image are separately shown in Figure 1a,b and are both grayscale images. For subsequent calculation, the software Matlab version R2020a was used to convert the processed image (Figure 1b) into a binary image of only black and white (Figure 1c) by using a Matlab image processing program coded by the author.

#### 2.3.2. Calculation of CF Cross-Section Perimeter and Area

Based on the binary image, Matlab was sequentially used to calculate the CF cross-section perimeter (C) and cross-section area (S). Firstly, the Canny Operator built into Matlab was used to extract the coordinate of each point on the boundary of the CF cross-section to generate a contour image, as shown in Figure 1d. The scale of the original SEM image and its number of pixels were used to calculate the actual size represented by each pixel. In this work, one pixel actually corresponded to 5 nm. Then, the numbers of pixels included in the perimeter and in the area of the CF cross-section were calculated by a binary image labeling algorithm. In the next step, the units of the perimeter and area were separately converted into μm and μm^2^. In order to better describe the shape of the CF cross-section, the roundness value α was used to describe the degree of how the CF cross-section was close to a perfect circle. The α value was calculated using Equation (2):

(2) $$\alpha =\frac{4\pi S}{C^{2}}$$

where *α* is the roundness value of CF, *S* is the cross-section area of CF and *C* is the cross-section perimeter of CF. According to Equation (2), the closer the CF cross-section shape is to a perfect circle, the closer α is to 1. When the CF cross-section shape is a perfect circle, *α* is equal to 1.

#### 2.3.3. Calculating CF Surface Groove Structure Parameters

**Figure 2:**
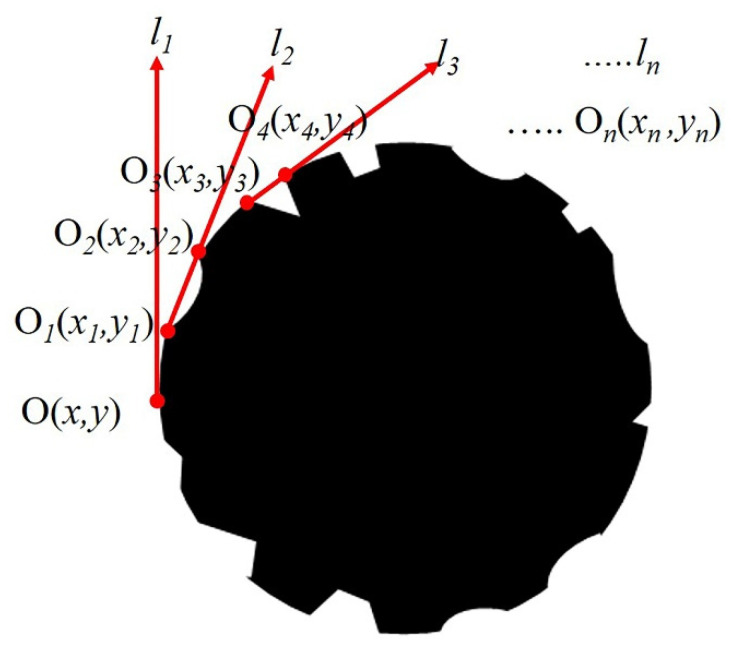
Schematic diagram of CF groove definition and calculation.

Due to the radial shrinkage of the original fiber in a coagulation bath during the CF manufacturing process, grooves are preserved. CF grooves as shown in Figure 1a can be mainly summarized into three types of ideal structural forms: trapezoidal groove, arcuate groove and triangular groove (as shown in Figure 2). No matter what structural forms the grooves are, they all open outwards. The distance between any two points on the edge of a groove is smaller than or equal to the opening distance of the groove. The opening distance was defined as the width of the groove. The deepest distance from the line between the furthest two points on the groove edge to the groove was defined as the depth of the groove. To calculate W and D, the linear equation tangent method was used to find out all grooves on the CF surface. The detailed methods are as follows.

Using another Matlab data analysis program coded by the author, the coordinate positions of every point on the CF cross-section edge were calculated and marked as *O*(*x*, *y*), as shown in Figure 2. All points on the edge of the CF cross-section edge could be classified into two categories: some on the smooth surface and the others on the groove surface. One random point was set as the origin point of the rectangular plane coordinate system and linked with other points. If all the other points were on one side of the line between this initial point and one point, this line was considered as a tangent line (l). The distance between these two points was defined as the width of one groove. The deepest distance between the tangent line and this groove was defined as the depth. All tangents and points of tangency that met these program criteria were found out and calculated, shown as *l*_1_, *l*_2_, *l*_3_ … *l*n, and *O*_1_(*x*_1_, *y*_1_), *O*_2_(*x*_2_, *y*_2_), *O*_3_(*x*_3_, *y*_3_) … *O*_n_ (*x*_n_, *y*_n_) in Figure 2. In the above calculation process, the Convhull function was used to achieve this process. The number of all grooves on the carbon fiber surface was recorded as N.

## 3. Results and Discussion

### 3.1. Cross-Section and Surface Morphology of CFs

**Figure 3:**
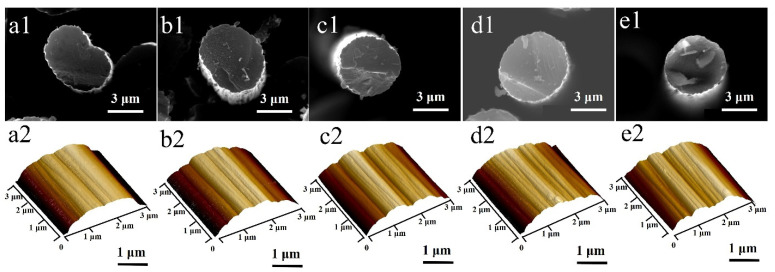
The SEM cross-section morphology image of: (**a1**) CF-1, (**b1**) CF-2, (**c1**) CF-3, (**d1**) CF-4, (**e1**) CF-5; and the AFM surface morphology image of: (**a2**) CF-1, (**b2**) CF-2, (**c2**) CF-3, (**d2**) CF-4, (**e2**) CF-5.

The cross-section morphologies of five types of CFs are shown in Figure 3a1–e1. These morphologies showed three kinds of shapes. The cross-section of CF-1 was like a cashew, CF-2 was like an oval and CF-3, CF-4 and CF-5 were close to a circle. The surface morphologies of five types of CFs are shown in Figure 3a2–e2, from which longitudinal grooves can be clearly observed. The Ra values of five types of CFs by AFM are listed in Table 1. The Ra of CF-1, CF-2 and CF-5 was bigger than CF-3 and CF-4.

### 3.2. Quantitative Characterization Results of CF Surface Microstructure

**Figure 4:**
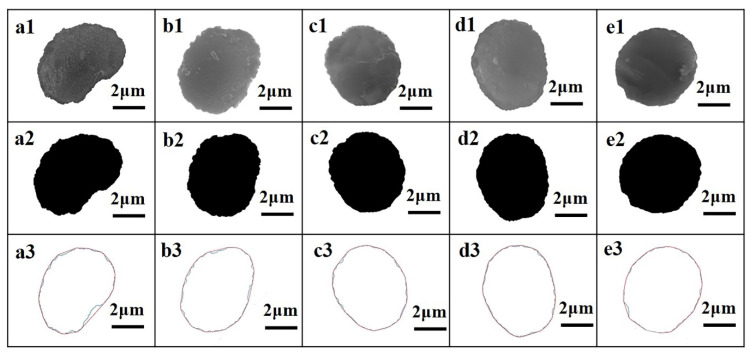
The cross-section grey image of: (**a1**) CF-1, (**b1**) CF-2, (**c1**) CF-3, (**d1**) CF-4, (**e1**) CF-5; the binary image of (**a2**) CF-1, (**b2**) CF-2, (**c2**) CF-3, (**d2**) CF-4, (**e2**) CF-5; the tangent image of: (**a3**) CF-1, (**b3**) CF-2, (**c3**) CF-3, (**d3**) CF-4, (**e3**) CF-5.

**Figure 5:**
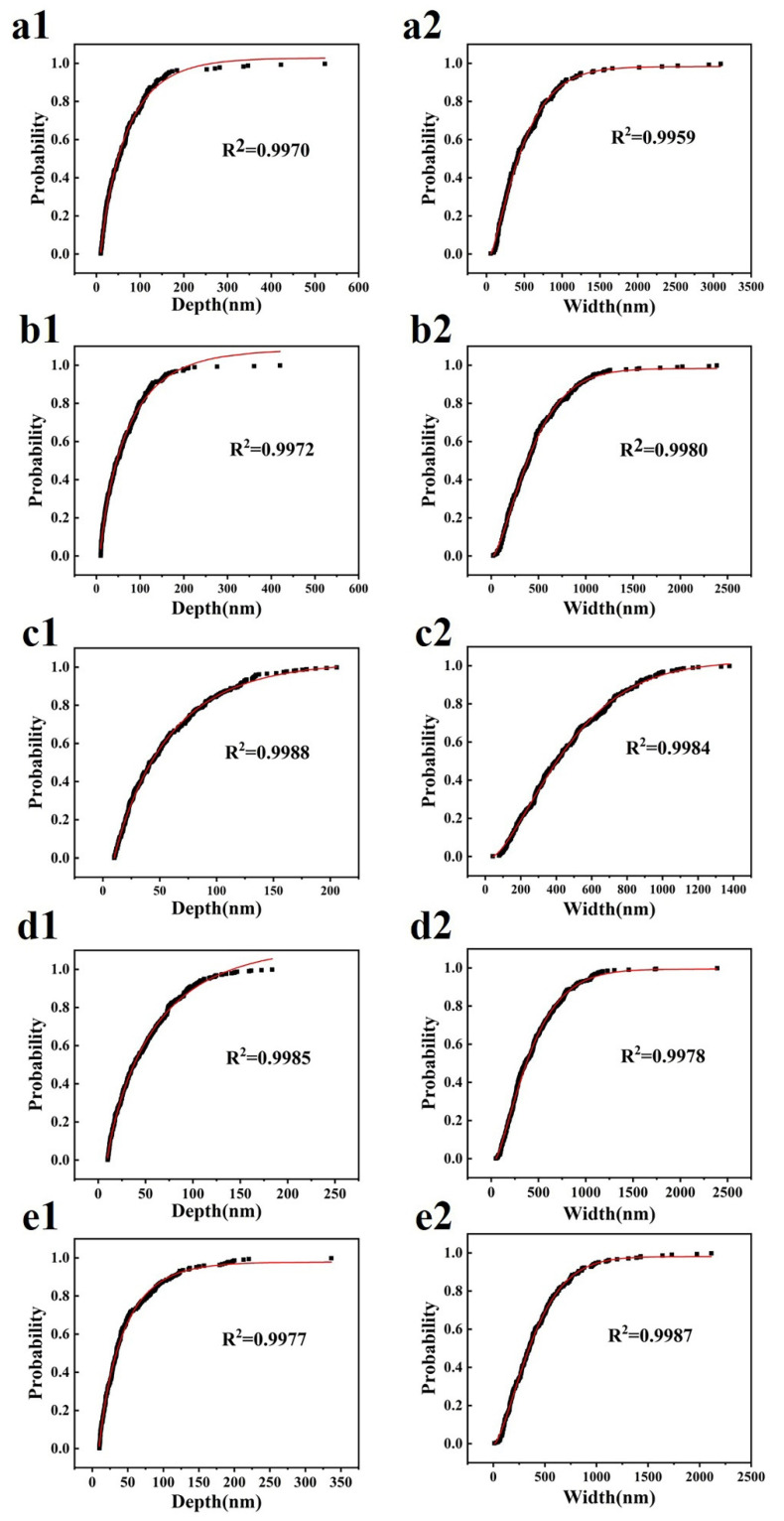
Weibull distribution fitting curve image of carbon fiber groove depth: (**a1**) CF-1, (**b1**) CF-2, (**c1**) CF-3, (**d1**) CF-4, (**e1**) CF-5; Weibull distribution fitting curve image of carbon fiber groove depth: (**a2**) CF-1, (**b2**) CF-2, (**c2**) CF-3, (**d2**) CF-4, (**e2**) CF-5.

**Figure 6:**
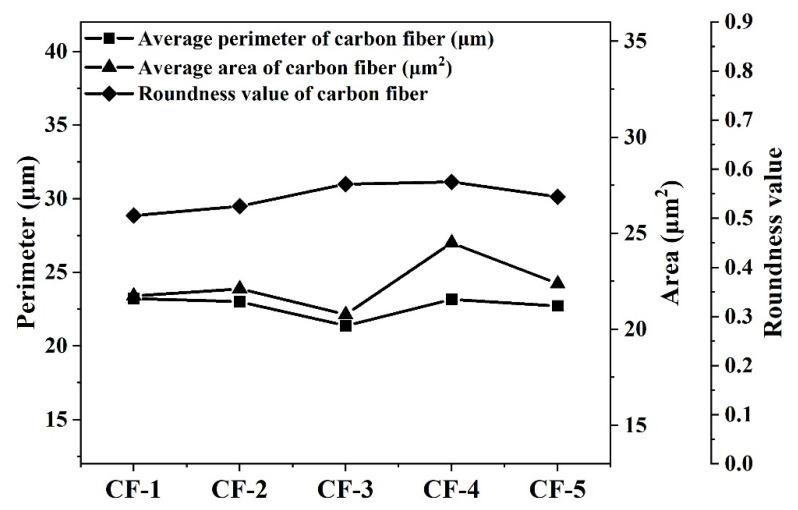
The average perimeter, area and roundness value of CFs.

Processed by the method of Section 2.1, Section 2.2 and Section 2.3, five types of CFs’ cross-section image results are shown in Figure 4. As the results show, the gray image of each CF cross-section was kept at the same profile to its binary image and tangent image. The detailed microstructure parameters of the CF cross-section are listed in Table 2. The groove depth and width of CFs were, respectively, fitted according to the Weibull distribution function. The fitting curve images are shown in Figure 5. Correlation coefficients of all fitted curves were bigger than 0.995. As shown in Figure 6, the average cross-section perimeter and average area of five types of CFs kept the same trend. Both the cross-section perimeter and area of CF-3 were the smallest in five types of CFs. The cross-section perimeter of CF-1 was the largest one, which was nearly equal to CF-4. Meanwhile, the cross-section area of CF-4 was the largest one. For roundness values, CF-1 was the smallest one in five types of CFs due to its cross-section like a cashew. The roundness of CF-2 was only a little bigger than CF-1. The roundness of CF-3, CF-4 and CF-5 was similar but bigger than CF-1 and CF-2, which was consistent with their cross-section shapes discussed in Section 3.1. The roundness of CF-4 was the largest, indicating that the cross-section shape of CF-4 was closest to a circle.

What needs be noted was that the perimeters of five types of CFs were between 21.38 μm (CF-3) and 23.23 μm (CF-1), which were quite different from their nominal perimeter of 16.3 μm (π*d*). This was mainly due to the fact that the calculation of the nominal perimeter of the CF cross-section refers to the calculation method of a standard circle, which ignored the grooves, bumps and pits on the CF surface. However, the areas of five types of CFs were between 20.77 μm^2^ and 24.51 μm^2^ and were nearly equal to their nominal area of 21.2 μm^2^ (0.25π*d*^2^). It is indicated that grooves, bumps and pits made a bigger difference to the CF cross-section perimeter than the area.

**Figure 7:**
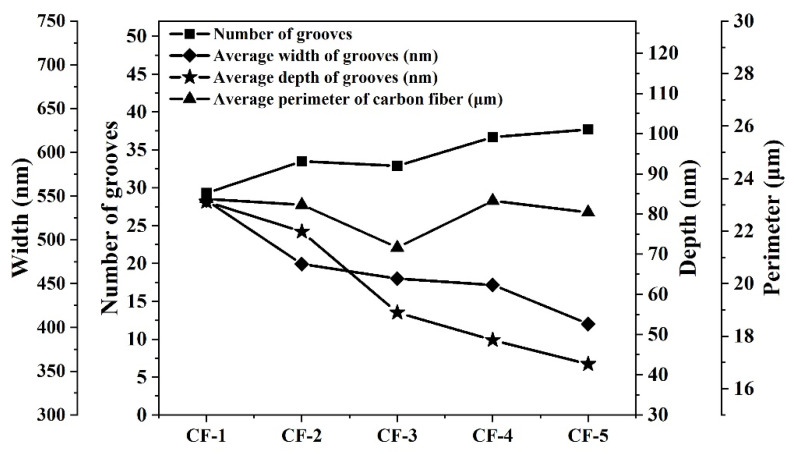
The relationship between groove parameters and average perimeter of CFs.

The average number of grooves on each single CF filament was between about 30 and 40, the average width was between about 400 nm and 550 nm and the average depth was between 40 nm and 85 nm. The D/W of five types of CFs was between 0.11 and 0.16. Further analysis for these groove data indicated a trend: from CF-1 to CF-5, the number of their grooves was increasing, while both the width and depth of their grooves were decreasing, as shown in Figure 7. The grooves on CF-1's surface were the widest and deepest, while the grooves on CF-5’s surface were the narrowest and shallowest. From Figure 7, there was no obvious correlation between the average perimeter and the groove parameters.

### 3.3. Groove Parameters and Surface Roughness of CFs

**Figure 8:**
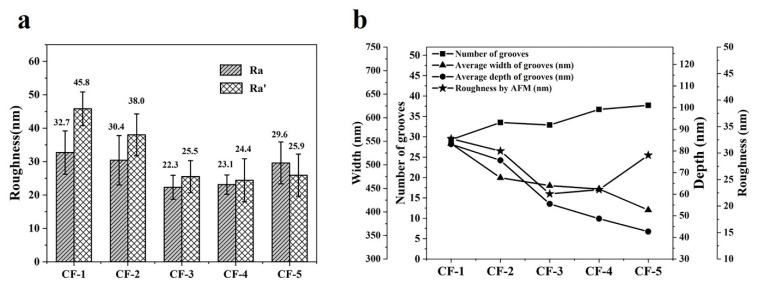
(**a**) Ra and Ra′ of CFs; (**b**) the relationship between groove parameters and surface roughness of CFs.

In order to further explore the relationship between groove parameters and Ra, a new calculation method was put forward to calculate a new roughness (Ra′). In this calculation method, all grooves on the CF surface were included, as shown in Equation (3):

(3) $${\mathrm{Ra}}^{\prime }=\frac{\sum_{i=1}^{n}\frac{1}{2}w_{i}d_{i}}{c}$$

where Ra′ is the new roughness of CF calculated by all grooves, *i* is the ith groove, *w_i_* is the width of the *i*th groove, *d_i_* is the ith groove and *C* is the perimeter of this CF filament. Ra and Ra′ are compared with each other in Figure 8a. It can be found that the Ra and Ra′ of CF-3, CF-4 and CF-5 were nearly equal to each other, while the Ra and Ra′ of CF-1 showed a big difference, as well as CF-2. The Ra′ of CF-1 and CF-2 was bigger than their Ra, which is due to their cashew and oval cross-section shapes. During the calculation of Ra′, all grooves around the side surface of CF were included, while during the calculation of Ra, only part of the side surface was included. For CF-1 and CF-2, some big grooves were inevitably missed in the calculation of Ra, which led to a smaller Ra. Though Ra was not totally equal to Ra′, they still showed highly similar trends, as shown in Figure 8a. Figure 8b shows the relationship between the groove parameters and surface roughness Ra of CF, showing that roughness had no obvious correlation with any single parameter of the grooves. Conversely, it could be inferred that roughness was decided by the number, width and depth of CFs together.

### 3.4. Specific Surface Area and Cross-Section Perimeter of CFs

**Figure 9:**
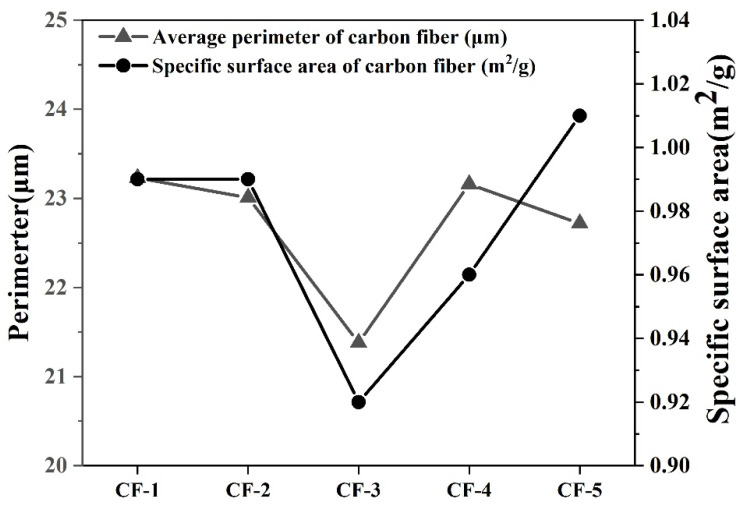
The relationship between specific surface area and perimeter of CFs.

Figure 9 shows the specific surface area and average perimeter of five types of CFs. The specific surface area of different CFs varied from about 0.90 m^2^/g to 1.00 m^2^/g. Figure 9 also shows the relationship between the specific surface area and average perimeter of CFs. The average perimeter of CFs was basically consistent with their specific surface area. Because the specific surface area was the total of all microstructures on the CF surface, this result indirectly proved that the calculation of perimeter by Matlab was accurate and reasonable.

### 3.5. Composite Interfacial Property and CF Surface Microstructure

**Figure 10:**
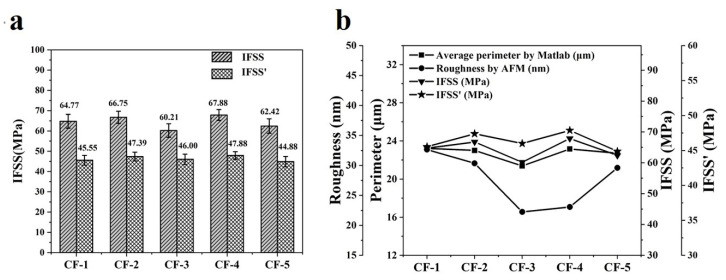
(**a**) IFSS and IFSS’ of CF composites and (**b**) the relationship between composite interfacial property, cross-section perimeter and surface roughness of CFs.

The test principle and calculation method of IFSS in Section 2.2 indicated that the values of IFSS were decided by the maximum debonding load F_max_, the diameter d of CF and the length L of carbon fibers embedded in resin. F_max_ and L were tested by the micro-droplet debonding test machine and they were relatively accurate, while the nominal perimeter (πd) had a large deviation with the realistic perimeter of the CF cross-section, which was already discussed in Section 3.2. Section 3.4 in this paper has already demonstrated that the calculation of perimeter by Matlab was accurate and reasonable. It is necessary to modify the IFSS by the realistic perimeter of CF. The modification method is shown in Equation (4):

(4) $${\mathrm{IFSS}}^{\prime }=\frac{\mathrm{IFSS}\times \pi d}{C}$$

where IFSS was the interfacial shear strength by the micro-droplet debonding test, IFSS’ was the modified value of IFSS, *d* was the diameter of CF and *C* was the average perimeter of CF. The diameter, chemical element ratio and composite interfacial properties of five kinds of CFs are listed in Table 3. As Figure 10a shows, the IFSS values of five types of CF composites were roughly equal to each other and were between 60 MPa and 68 MPa. The IFSS’ values of five types of CF composites were roughly equal to each other and were between 44 MPa and 48 MPa. The percentage differences between IFSS’ and IFSS were as high as 23% to 30%. Because all grooves on the CF surface were included in the calculation of the CF cross-section perimeter, IFSS’ should be a more accurate parameter to reflect the real composite interfacial property than IFSS.

The composite interfacial property is affected by both the physical structure and chemical property of CFs. In this study, the chemical properties of five types of CFs were controlled to be the same, which could be proved by the nearly same chemical element ratio of O and C (O/C), as shown in Table 3. Figure 10b shows the relationship of average perimeter by Matlab, roughness by AFM, IFSS and IFSS’. It could be seen that IFSS, IFSS’ and the average perimeter of CFs all showed the same trend. However, this trend was different from the roughness of CFs.

### 3.6. Interface Bonding Mechanism of CF Composites

**Figure 11:**
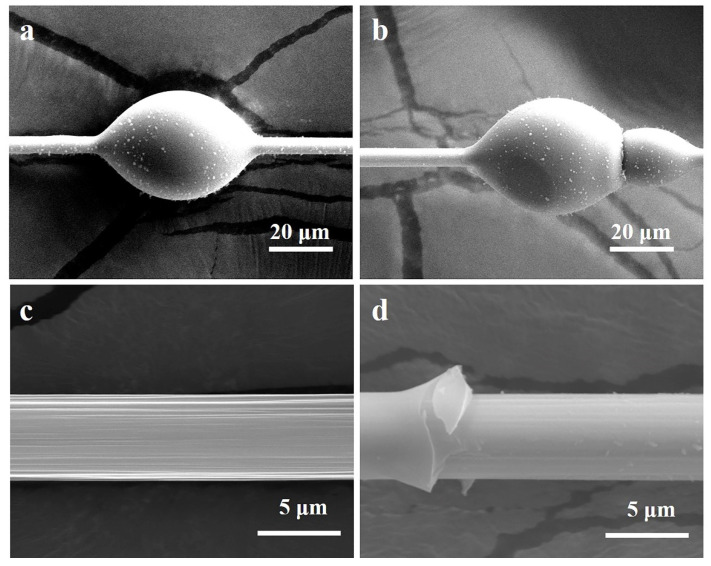
Micro-droplet debonding test sample image of: (**a**) before fracture, (**b**) after fracture, (**c**) CF surface morphology, (**d**) fracture morphology of CF/EP composite.

Figure 11a,b are, respectively, the micro-droplet debonding test sample image before and after debonding. The micro-sphere was peeled in a complete status, indicating that the debonding occurred mainly in the interface of the CF/Epoxy(EP) composite. Figure 11c,d show the CF surface morphology in the initial status and the CF surface after debonding. After the micro-sphere was peeled, the grooves on CF were still preserved, but they became shallower. This indicated that some resin was filled in the grooves and preserved.

**Figure 12:**
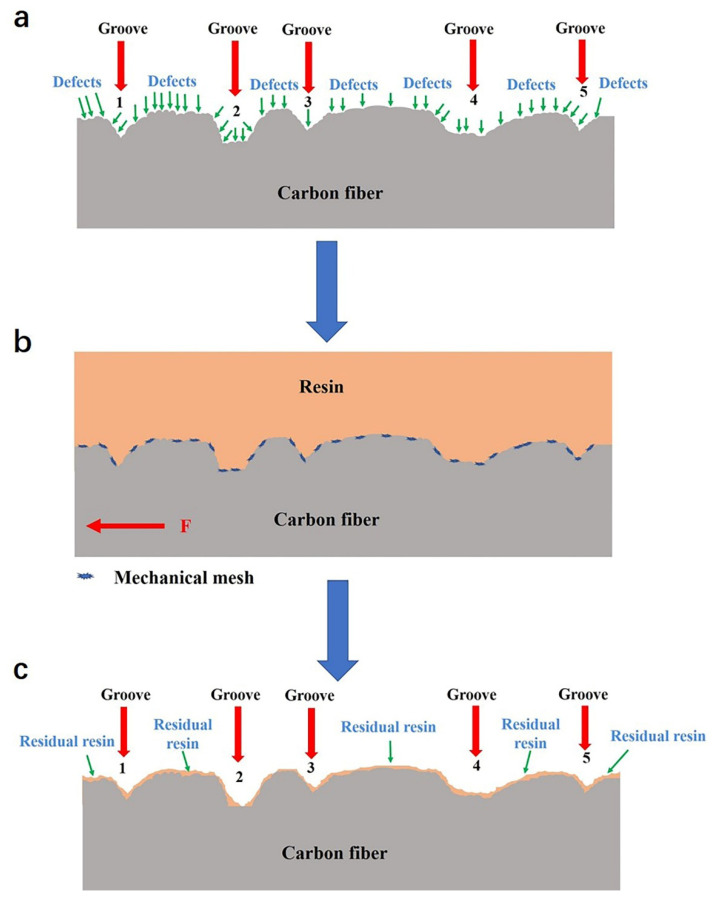
Schematic diagram of: (**a**) CF surface microstructure, (**b**) composite interface structure, (**c**) composite fracture morphology.

For CFs with the same chemical activities, the composite interface property is mainly affected by the physical structure [37]. In this paper, a composite interface bonding mechanism based on defect capture was put forward to explain the result that both IFSS and IFSS’ kept a strong consistency with the perimeter or specific surface area in Section 3.5. This mechanism is shown in Figure 12. The CF surface microstructure contains not only the grooves (tens of nm or even bigger), but also lots of defects (a few nm or smaller), as shown in Figure 12a. In fact, a lot of defects, such as gaps, uneven microstructures, micro cracks, punctures and diametrical bulges, are on the CF surface [38,39,40]. These defects are much smaller than grooves, but their number is much more than the number of grooves. During the process of composite interface formation, resin flowed into grooves and covered the CF surface. Lots of resin molecules were captured by these defects to form a mechanical mesh (Figure 12b). As Figure 12c shows, when the composite interface was destroyed under shear stress, large amounts of resin were peeled from the CF surface, while small amounts of resin were captured by the defects on the CF surface.

## 4. Conclusions

An image quantitative characterization technique for carbon fiber surface microstructures was established in this article. The cross-section perimeter and area of CFs and the groove parameters (the number, width and depth of grooves) were all calculated by this technique. A new calculation method of roughness based on groove parameters was put forward and proved to be feasible. The result demonstrated that roughness values were more significantly affected by larger grooves. The roughness of the CF surface was proved to be not strongly related to any single groove parameter. The specific surface area of CFs showed a consistent trend with their average perimeters, which indirectly proved that the calculation of perimeter by Matlab was accurate and reasonable.

Based on the realistic perimeter of CFs, a modification method of IFSS was also put forward to reflect the real composite interfacial property. It was the CF perimeter calculated by Matlab that was demonstrated to have a strong consistent trend with the composite interfacial property, not the CF surface roughness. This result may provide some new ideas to improve the CF composite interface property. In the end, a composite interface bonding mechanism based on defect capture was put forward, which may help researchers better understand and establish interface bonding theories.

## Figures and Tables

**Table 1:** The surface roughness of 5 types of CFs.

Samples	CF-1	CF-2	CF-3	CF-4	CF-5
Roughness (Ra, nm)	32.7 ± 6.5	30.4 ± 7.4	22.3 ± 3.6	23.1 ± 2.9	29.6 ± 6.3

**Table 2:** Surface microstructure parameters of 5 types of CFs.

Samples	Perimeter (C, μm)	Area (S, μm^2^)	Roundness Value (α)	Number (N)	Width (W, nm)	Depth (D, nm)	Depth/Width (D/W)
CF-1	23.23 ± 1.30	21.74 ± 1.20	0.506	29.3 ± 2.8	545.05	83.08	0.15
CF-2	23.01 ± 1.29	22.11 ± 1.51	0.525	33.5 ± 3.4	472.62	75.64	0.16
CF-3	21.38 ± 0.86	20.77 ± 1.01	0.570	32.9 ± 3.9	455.82	55.50	0.12
CF-4	23.16 ± 1.05	24.51 ± 1.85	0.574	36.7 ± 4.8	448.69	48.66	0.11
CF-5	22.72 ± 0.98	22.38 ± 1.59	0.544	37.7 ± 5.0	404.07	42.67	0.11

**Table 3:** Surface physicochemical properties and composite interfacial property of CFs.

Samples	Diameter (d, μm)	Chemical Element Ratio (O/C)	Composite Interfacial Property		
			IFSS (MPa)	IFSS’ (MPa)	Percentage Difference (%)
CF-1	5.2	0.07	64.77 ± 3.42	45.55 ± 2.40	−29.68
CF-2	5.2	0.08	66.75 ± 3.00	47.39 ± 2.13	−29.01
CF-3	5.2	0.08	60.21 ± 3.33	46.00 ± 2.54	−23.59
CF-4	5.2	0.06	67.88 ± 2.67	47.88 ± 1.88	−29.47
CF-5	5.2	0.07	62.42 ± 3.56	44.88 ± 2.56	−28.10

## Data Availability

The data presented in this study are available on request from the corresponding author.
